# Misclassification of malaria as pneumonia in children in sub-Saharan Africa

**DOI:** 10.1093/ije/dyaf040

**Published:** 2025-04-10

**Authors:** Christian Bottomley, Alice Kamau, Juliet O Awori, Amanda J Driscoll, Daniel E Park, Samba O Sow, Milagritos D Tapia, Karen L Kotloff, Bernard E Ebruke, Martin Antonio, Stephen R C Howie, Richard J Hayes, J Anthony G Scott

**Affiliations:** International Statistics and Epidemiology Group, London School of Hygiene & Tropical Medicine, London, United Kingdom; Department of Infectious Disease Epidemiology and International Health, London School of Hygiene & Tropical Medicine, London, United Kingdom; KEMRI-Wellcome Trust Research Programme, Kilifi, Kenya; KEMRI-Wellcome Trust Research Programme, Kilifi, Kenya; Center for Vaccine Development and Global Health, University of Maryland School of Medicine, Baltimore, United States; Department of Environmental and Occupational Health, The George Washington University, Washington, DC, United States; Centre pour le Développement des Vaccins, Bamako, Mali; Center for Vaccine Development and Global Health, University of Maryland School of Medicine, Baltimore, United States; Center for Vaccine Development and Global Health, University of Maryland School of Medicine, Baltimore, United States; MRC Unit The Gambia at the London School of Hygiene and Tropical Medicine, Fajara, The Gambia; International Foundation Against Infectious Disease in Nigeria (IFAIN), Abuja, Nigeria; MRC Unit The Gambia at the London School of Hygiene and Tropical Medicine, Fajara, The Gambia; MRC Unit The Gambia at the London School of Hygiene and Tropical Medicine, Fajara, The Gambia; Department of Paediatrics, University of Auckland, Auckland, New Zealand; College of Medicine, Nursing and Health Sciences, Fiji National University, Suva, Fiji; International Statistics and Epidemiology Group, London School of Hygiene & Tropical Medicine, London, United Kingdom; Department of Infectious Disease Epidemiology and International Health, London School of Hygiene & Tropical Medicine, London, United Kingdom; Department of Infectious Disease Epidemiology and International Health, London School of Hygiene & Tropical Medicine, London, United Kingdom; KEMRI-Wellcome Trust Research Programme, Kilifi, Kenya; The Nuffield Department of Medicine, Oxford University, Oxford, United Kingdom

**Keywords:** pneumonia, malaria, misclassification

## Abstract

**Background:**

The World Health Organization (WHO) clinical case definitions for pneumonia were designed to prioritize sensitivity over specificity. In sub-Saharan Africa, the disease that is most likely to be misclassified as pneumonia is *Plasmodium falciparum* malaria.

**Methods:**

By using chest X-ray positivity as an indicator for pneumonia, we estimated the extent of pneumonia misclassification due to malaria in the Pneumonia Etiology Research for Child Health (PERCH) study. Additionally, we developed a simple model to predict the proportion of pneumonia cases as defined by the WHO that could be attributed to malaria in settings with varying levels of malaria parasitaemia prevalence.

**Results:**

In the PERCH study, the prevalence of malaria parasitaemia was low (4.7% among WHO pneumonia cases and 1.4% among controls) and we estimate that only 2.5% of WHO pneumonia cases were misclassified. However, when assuming a prevalence of malaria parasitaemia of 24%, corresponding to the average for malaria-endemic areas in Africa, we estimate that 28% of WHO pneumonia cases are misclassified. Among malaria-slide-positive WHO pneumonia cases in PERCH, lower chest wall indrawing [adjusted odds ratio (aOR) =18.1, 95% confidence interval (95% CI): 1.9, 175.8, *P *=* *0.012], crackles on chest auscultation (aOR = 13.1, 95% CI: 1.4, 127.4, *P *=* *0.027), and nasal flaring (aOR = 5.9, 95% CI: 1.1, 32.8, *P *=* *0.041) were associated with chest X-ray positivity.

**Conclusion:**

In settings that are typical of sub-Saharan Africa, we predict that one-quarter of WHO-defined pneumonia cases are malaria rather than pneumonia. Among children with WHO pneumonia who also test positive for malaria parasitaemia, clinical features that favour pneumonia include lower chest wall indrawing, nasal flaring, and crackles on chest auscultation.

Key Messages

- There is a significant overlap in symptoms between malaria and pneumonia, which can lead to the misdiagnosis of both conditions.
- In malaria-endemic settings in sub-Saharan Africa, as many as one in four cases of clinical pneumonia, as defined by the World Health Organization, is misdiagnosed due to malaria.
- Certain clinical features such as lower chest wall indrawing, nasal flaring, and crackles on chest auscultation indicate pneumonia in children with malaria parasitaemia.

## Introduction

In 2019, pneumonia and malaria were estimated to cause 14% and 7.1%, respectively, of all childhood deaths worldwide [1–3]. In malaria-endemic countries in sub-Saharan Africa, a large proportion of acute febrile illnesses in children are caused by malaria or pneumonia. Differentiating between these causes is challenging, particularly in primary care, in which diagnoses rely entirely on symptoms and clinical signs [4–9]. For many years, febrile illness has been treated presumptively as malaria whereas cough/breathing difficulty accompanied by fast breathing has been treated as pneumonia [10–12].

Following clinical research in the 1980s and 1990s, the World Health Organization (WHO) derived a three-category clinical case definition for pneumonia that applied to children with cough or difficulty breathing [13]. Non-severe pneumonia was defined by fast breathing, severe pneumonia by lower chest wall indrawing, and very severe pneumonia by the presence of at least one danger sign (i.e. central cyanosis, inability to breastfeed/drink, vomiting everything, convulsions, lethargy, unconsciousness, or severe respiratory distress). In 2013, the WHO revised the classification of pneumonia, using the same signs, into two categories: non-severe and severe. Under this revised classification, the condition that was formerly considered ‘very severe pneumonia’ was reclassified as ‘severe pneumonia’, and ‘severe pneumonia’ and ‘non-severe pneumonia’ were combined into a single category of ‘non-severe pneumonia’ [14].

In developing the original pneumonia case definitions, the WHO prioritized sensitivity to ensure that no child who might benefit from antibiotics is denied treatment [15]. However, the trade-off for high sensitivity was poor specificity: many children who do not have pneumonia are also captured by the case definition. In sub-Saharan Africa, the disease that is most likely to be misclassified as pneumonia is *Plasmodium falciparum* malaria [11, 16]. At an average prevalence of 24%, malaria infection is common in the region [17] and frequently associated with respiratory symptoms [12, 18, 19]. Although the mechanism that links malaria with respiratory symptoms is unclear, it is thought that metabolic acidosis, which is common in malaria patients and often compensated for by deep breathing, could provide an explanation [6].

Although malaria infection is a potential source of misclassification, not all malaria-slide-positive WHO pneumonia cases are misclassified: genuine coinfections can also occur. In fact, they may be common in malaria-endemic areas, as there is evidence to suggest that malaria infection increases the risk of coinfection with a respiratory pathogen [20, 21].

Thus, the extent to which pneumonia misclassification occurs under the WHO criteria is uncertain [15]. However, data from chest X-rays present a possible avenue for gauging this misclassification. Here, we use data on chest X-ray (CXR) positivity that were collected as part of the Pneumonia Etiology Research for Child Health (PERCH) study [22, 23]—a multi-country case–control study of pneumonia aetiology—to estimate the proportion of WHO-defined pneumonia that is misclassified. Additionally, we describe the correlation between the WHO danger signs and CXR-positivity, and develop a simple model to predict misclassification in settings that are characterized by different levels of malaria parasitaemia prevalence.

## Methods

### Study sites and population

PERCH was a case–control study that was conducted between August 2011 and January 2014 in seven low- and middle-income countries in sub-Saharan Africa and Asia. This analysis is restricted to three sites that were malaria-endemic, i.e. Kilifi, Kenya; Basse, The Gambia; and Bamako, Mali. No malaria was identified in South Africa, Thailand, or Bangladesh and only two cases of malaria were identified in Zambia. Details on the collection of clinical data and laboratory tests are provided in the Supplementary material and the full study protocol is available at https://publichealth.jhu.edu/ivac/resources [24]. Briefly, cases comprised children who were aged 1–59 months and hospitalized with severe or very severe pneumonia following the (pre-2013) WHO definitions [13, 25]. Children who met the criteria for non-severe pneumonia were not included, as the focus of PERCH was on hospitalized pneumonia, which tends to be severe [25]. CXRs were performed on all cases, with CXR-positivity determined by evidence of alveolar consolidation or any other infiltrate on a chest radiograph that was performed ≤72 hours after presentation [26, 27] (more detail on CXR classification is provided in table 1 of Cherian *et al.* [26]). The controls were children who were aged 1–59 months, randomly selected from the catchment area for cases, and did not meet the WHO criteria for severe or very severe pneumonia [28]. Controls were frequency-matched to cases by enrolment date and age group.

### Estimation of positive predictive value of WHO-defined pneumonia in PERCH

To estimate the positive predictive value (PPV) of the WHO pneumonia definition, we used CXR results from PERCH. Additionally, we made three assumptions:

- The WHO definition of pneumonia is 100% specific among malaria-slide-negative hospital admissions in children aged 1–59 months.
- CXR-positivity is 100% specific for true pneumonia.
- The sensitivity of CXR findings is independent of the presence of malaria parasitaemia.

A formula for PPV was derived by classifying the WHO pneumonia cases into four groups based on the *unobserved* true pneumonia status (n) and observed malaria-slide result (m):

Group 0: Double negative (*n*^–^, *m*^–^)

Group 1: Malaria-slide-negative (*n*^+^, *m*^–^)

Group 2: Coinfected (*n*^+^, *m*^+^)

Group 3: Misclassified (*n*^–^, *m*^+^)

Let $p_{i}$ denote the proportion of WHO pneumonia cases in Group i (i=0,1,2, 3). The PPV can then be expressed as the sum of $p_{1}$ and $p_{2}$:


(1) $$\mathrm{PPV}\;=p_{1}+p_{2}.$$

Given the assumption of 100% specificity of the WHO criteria among malaria-slide negatives, we expect all malaria-slide negatives to be in Group 1 and none in Group 0. This implies that $p_{0}=0$ and allows us to estimate $p_{1}$ as the proportion of malaria-slide negatives among the WHO pneumonia cases. In other words, $p_{1}=1-v$, where v is the proportion of malaria-slide-positive cases among the WHO pneumonia cases.

To estimate the coinfected proportion, $p_{2}$, we make use of the CXR data. Let q denote the proportion of WHO pneumonia cases that are both CXR-positive and malaria-slide-positive, and let r denote the proportion that are both CXR-positive and malaria-slide-negative. We estimate $p_{2}$ as q multiplied by the factor (1-v)/r to account for the imperfect sensitivity of CXR. Note that r/(1-v) corresponds to the proportion that are CXR-positive in the malaria-slide-negative group (Group 1). Thus, as this group are true pneumonia cases by assumption 1, we are inflating q by 1/(CXR sensitivity).

Having obtained estimates $p_{1}$ and $p_{2}$, we estimate PPV from Equation 1: 


(2) $$\mathrm{PPV}\;=(1-v)+q\times \frac{\left(1-v\right)}{r}.$$

**Figure 1.**
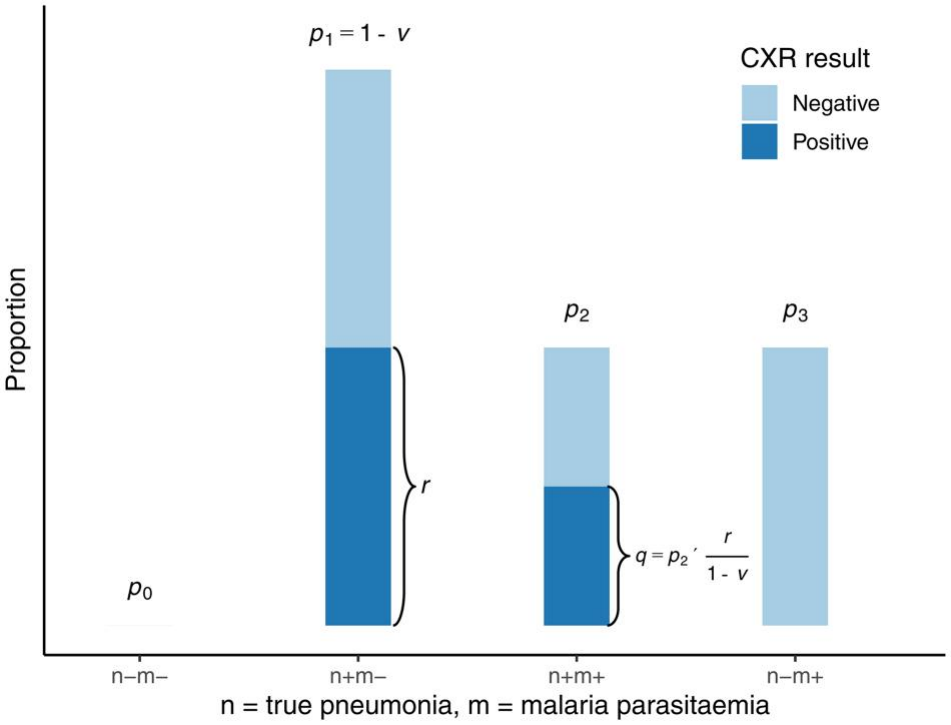
Schematic of the method used to estimate the PPV of clinical pneumonia as defined by the WHO. WHO-defined cases are classified into four groups that are defined by true pneumonia and malaria-slide result, with the proportion in each group denoted $p_{i}\;(i=0,1,2,3)$. The sum of the middle two bars ($p_{1}+p_{2}$) represents the proportion of WHO-defined clinical cases that are true cases of pneumonia (PPV), whereas the last bar ($p_{3}$) represents the misdiagnosed proportion. Because true pneumonia status is unknown, the direct estimation of $p_{i}\;$is not possible. Instead, we use three observable quantities: (i) the proportion, v, of WHO cases that are malaria-slide-positive; (ii) the proportion, q, of WHO cases that are both positive on CXR and malaria-slide-positive; and (iii) the proportion, r, of WHO cases that are both CXR-positive and malaria-slide-negative. First, we estimate $p_{1}$ by assuming that the WHO pneumonia criteria are 100% specific among malaria-slide negatives. This implies that $p_{0}=0$ and $p_{1}=1-v$. Then, to estimate $p_{2}$, we divide q by an estimate of the sensitivity of CXR ($\frac{r}{1-v}$) such that $p_{2}=\;q\times \frac{\left(1-v\right)}{r}$. Here, we assume that the sensitivity of CXR is independent of malaria parasitaemia. Finally, using the fact that the probabilities sum to one, $p_{3}$ can be estimated as $p_{3}=1-p_{1}-p_{2}$.

The derivation of this formula is depicted graphically in Figure 1 and a formal derivation is provided in the Supplementary information.

### Modelling the relationship between PPV and malaria parasitaemia

We obtained PPV estimates for settings with different levels of malaria parasitaemia prevalence by making use of our estimates of $p_{i}$ from the PERCH cases and data on the prevalence of malaria from the PERCH controls. We used the latter as an estimate of the prevalence of malaria parasitaemia at the PERCH sites.

Let λ denote the average rate of WHO pneumonia across the PERCH sites and u denote the prevalence of malaria parasitaemia in the PERCH controls. Among members of the PERCH communities that were negative for malaria parasitaemia, the expected rate of malaria-slide-negative WHO pneumonia cases (Group 1) is $\lambda p_{1}/(1-u$). Similarly, among community members that were positive for malaria parasitaemia, the expected rates of coinfected (Group 2) and misclassified (Group 3) WHO pneumonia cases are ${\lambda p}_{2}/u$ and ${\lambda p}_{3}/u$, respectively.

Based on these rates, in a setting in which the prevalence of malaria parasitaemia is $u^{*}$, the PPV is given by:


(3) $$\begin{array}{c} \mathrm{PPV}=\frac{rate\;of\;true\;pneumonia}{rate\;of\;WHO\;pneumonia}\\ \begin{array}{c} =\frac{\lambda p_{1}(1-u^{*})/(1-u)+{\lambda p}_{2}u^{*}/u}{\lambda p_{1}(1-u^{*})/(1-u)+{\lambda p}_{2}u^{*}/u+{\lambda p}_{3}u^{*}/u}\\ =\frac{p_{1}(1-u^{*})/(1-u)+p_{2}u^{*}/u}{p_{1}(1-u^{*})/(1-u)+p_{2}u^{*}/u+p_{3}u^{*}/u}. \end{array} \end{array}$$

We used this formula to predict PPV at various levels of malaria parasitaemia, assuming the same rate of true pneumonia as in PERCH. As a sensitivity analysis, we also computed PPV at rates of true pneumonia that were 0.5 and 2 times greater than the PERCH rate.

We also used the model to estimate the fraction of WHO pneumonias that were caused by malaria infection, i.e. the population-attributable fraction. Specifically, we used the model to predict the coinfected proportion and then multiplied that by an estimate of the attributable fraction (AF). To compute the AF, we calculated the odds ratio (OR) by comparing the prevalence of malaria parasitaemia between CXR-positive cases and community controls, and used the standard formula AF=1-1/OR.

## Results

### Characteristics of WHO-defined pneumonia cases

**Figure 2.**
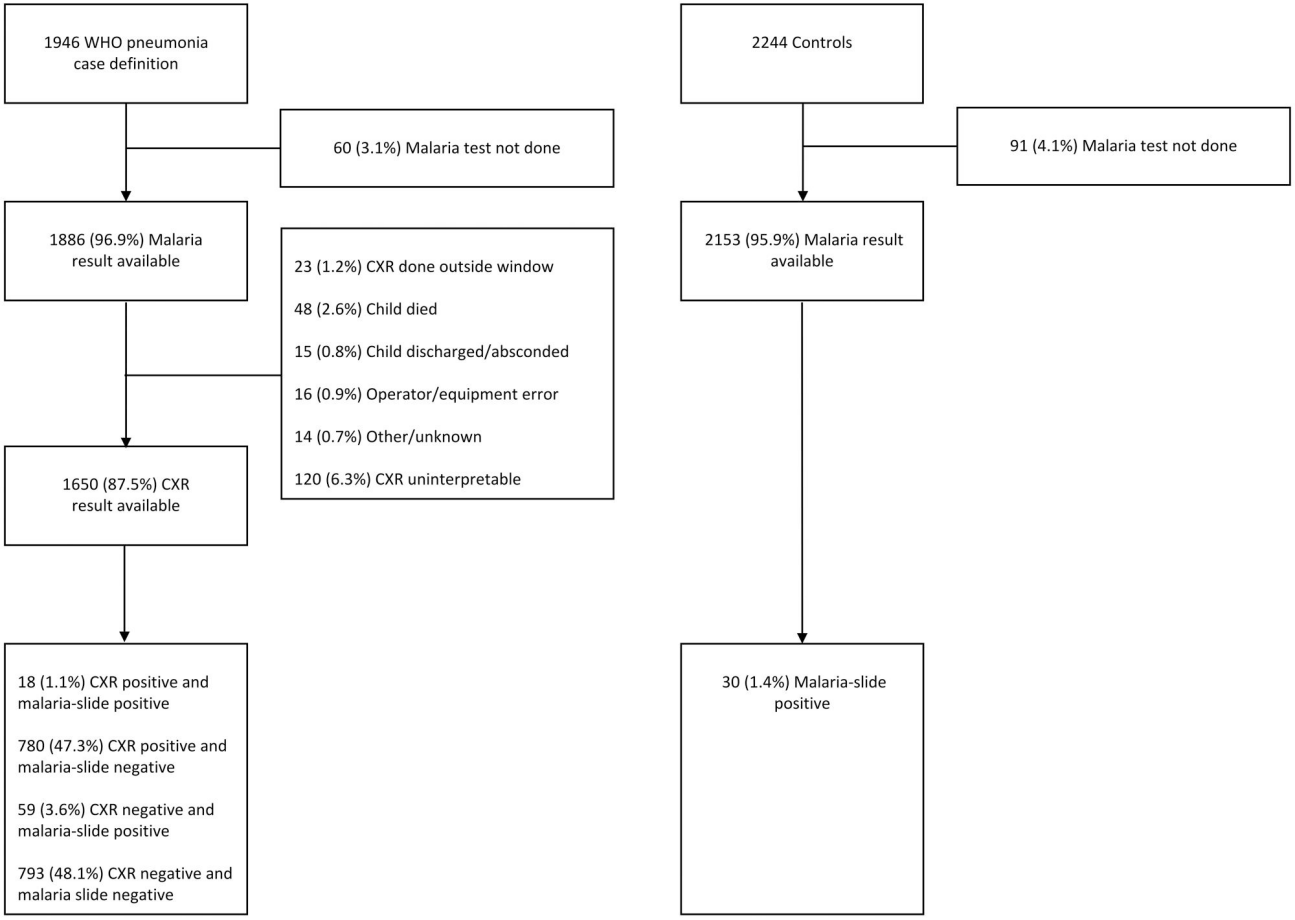
Breakdown of children included in the analysis and reasons for exclusion.

A total of 1946 cases of severe or very severe pneumonia following the (pre-2013) WHO definitions [13, 25] and 2244 controls were enrolled in the three PERCH sites in which malaria was detected (Figure 2). Meanwhile, 1650 cases had both a CXR result and a malaria result, with 798 (48.4%) exhibiting radiological signs of pneumonia and 77 (4.7%) testing positive for malaria parasitaemia. Among those with a positive CXR result, 204 had consolidation only, 435 had other infiltrates without consolidation, and 159 had both; 18 cases had both radiological pneumonia and malaria infection. The clinical characteristics of the cases are shown in Table 1. Among 2153 community controls with available malaria results, 30 (1.4%) had a positive slide: 15/814 (1.8%) in Kenya, 7/619 (1.1%) in The Gambia, and 8/720 (1.1%) in Mali.

**Table 1.** Characteristics of 1650 patients with clinically defined pneumonia and complete data on malaria slide and CXR results.

	Malaria-slide-positive				Malaria-slide-negative			
	CXR+ (*n* = 18)		CXR– (*n* = 59)		CXR+ (*n* = 780)		CXR– (*n* = 793)	
Female (%)	56		42		41		40	
Age in months [median (range)]	22	(12, 35)	28	(12, 44)	9	(4, 17)	8	(3, 17)
Weight-for-age z-score [median (range)]	–1.38	(–2.23, –0.74)	–1.60	(–2.41, –0.73)	–1.54	(–2.64, –0.5)	–1.15	(–2.19, –0.05)
Haemoglobin [median (range)]	7.8	(6.2, 9.9)	8.6	(5.7, 9.9)	9.7	(8.5, 10.6)	9.9	(9.0, 10.9)
White cell count [median (range)]	13.0	(8.6, 15.2)	10.6	(7.6, 14.3)	13.5	(9.5, 17.4)	11.8	(8.8, 15.8)
% Neutrophils [median (range)]	46.1	(32.0, 53.3)	52.6	(37.7, 66.0)	48.0	(33.9, 61.7)	46.4	(32.2, 61.1)

CXR+, chest-X-ray-positive; CXR–, chest-X-ray-negative. Haemoglobin is given in grams per decilitre. White cell count is measured as thousands of white cells per microlitre.

### Factors associated with CXR-positivity in malaria-slide-positive WHO-defined pneumonia cases

The spectrum of clinical signs and symptoms when CXR-positive (*n* = 18) and CXR-negative (*n* = 59) malaria-slide-positive WHO-defined pneumonia cases are compared is shown in Supplementary Table S1 (see online supplementary material for a colour version of this table). The most important associations with CXR-positivity were, in rank order: lower chest wall indrawing [adjusted odds ratio (aOR) =18.1, 95% confidence interval (95% CI): 1.9, 175.8, *P *=* *0.012], crackles on chest auscultation (aOR = 13.1, 95% CI: 1.4, 127.4, *P *=* *0.027), and nasal flaring (aOR = 5.9, 95% CI: 1.1, 32.8, *P *=* *0.041). CXR-positivity was negatively associated with multiple or prolonged convulsions (aOR = 0.14, 95% CI: 0.02, 0.95, *P *=* *0.044).

### PPV of WHO-defined clinical pneumonia

By applying the three assumptions outlined in the Methods and Figure 1, we estimated that, among PERCH cases that met the WHO pneumonia criteria, 95.3% were malaria-slide-negative (and true pneumonia cases), 2.2% were coinfected (malaria-slide-positive with pneumonia), and 2.5% were misclassified (malaria-slide-positive without pneumonia). Therefore, the PPV was calculated as 97.5% (95.3% + 2.2%). The calculated PPV was slightly lower in children aged ≥1 year (95.4%) and in cases of very severe WHO pneumonia (93.4%) whereas, for malaria-slide-positive cases, it was considerably lower, at 47.1% (Table 2).

**Table 2.** PPV of clinical pneumonia, as defined by the WHO, in the Pneumonia Etiology Research for Child Health study.

		Malaria-slide-positive				Malaria-slide-negative				Proportion of WHO pneumonia cases correctly classified (PPV)			
										Malaria-slide-positive cases		All cases	
	*N*	All	**%** ^a^	CXR+	**%** ^b^	All	%	CXR+	**%** ^c^	**%** ^d^	**95% CI** ^e^	**%** ^f^	**95% CI** ^g^
All cases	1650	77	4.7	18	1.1	1573	95.3	780	47.3	47.1	25.5–68.8	97.5	96.5–98.5
Severe pneumonia	1026	15	1.5	9	0.9	1011	98.5	539	52.5	100^h^		100^h^	
Very severe pneumonia	624	62	9.9	9	1.4	562	90.1	241	38.6	33.9	11.9–55.8	93.4	91.2–95.6
<1 year	985	19	1.9	4	0.4	966	98.1	471	47.8	43.2	0.9–85.4	98.9	98.1–99.7
≥1 year	665	58	8.7	14	2.1	607	91.3	309	46.5	47.4	22.8–72.0	95.4	93.3–97.6

a Estimate of  vin  Equation 2.

b Estimate of  qin  Equation 2.

c Estimate of r in Equation 2.

d Estimated as q(1-v)/vr.

e CI obtained from the CI for q and neglecting uncertainty in the estimates of r and v.

f Estimated as 1-v+q(1-v)/r.

g CI obtained from the CI for q and neglecting uncertainty in the estimates of r and v.

h Truncated at 100% because estimate exceeds 100%.

CXR+, chest X-ray-positive.

**Figure 3.**
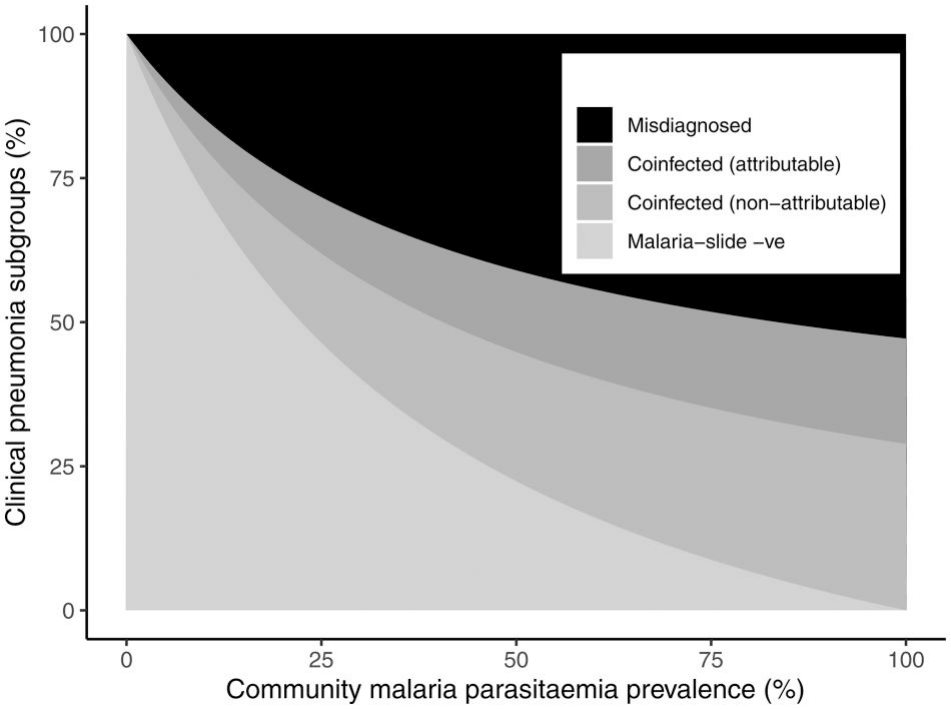
Misclassification of clinical pneumonia cases, as defined by the WHO, as a function of the community prevalence of malaria parasitaemia in children aged 1–59 months. Cases are categorized as either: (i) malaria-slide-negative, (ii) coinfected and attributable to malaria, (iii) coinfected and not attributable to malaria, and (iv) misdiagnosed. The PPV of the WHO pneumonia definition corresponds to the proportion of cases that are either malaria-slide-negative or coinfected.

Based on our model, we estimate that, in a region with a typical malaria parasitaemia prevalence of 24% [17], 47.8% of WHO pneumonia cases are malaria-slide-negative, 24.6% are coinfected, and 27.6% are misclassified (Figure 3). This corresponds to a PPV of 72.4%. Among the coinfected group, we estimate that the proportion that is attributable to malaria is 38.8%. Therefore, the proportion of all WHO pneumonia cases that is attributable to malaria is 9.5% (38.8% of the coinfected proportion). From our sensitivity analysis, we estimate that a true pneumonia incidence of twice the PERCH incidence increases the PPV to 83.9% and an incidence of half the PERCH incidence decreases the PPV to 56.7% (Supplementary Figure S1; see online supplementary material for a colour version of this figure).

## Discussion

Among clinically diagnosed WHO pneumonia cases from the PERCH study, we estimated that 97.5% were true instances of pneumonia and 2.5% were misclassified. This high PPV is largely attributable to the low prevalence of malaria parasitaemia in the PERCH WHO pneumonia cases. Indeed, in settings that are more representative of malaria endemicity, we estimate the PPV to be in the region of 70%.

As countries progress towards malaria elimination, we predict that there will be significant declines in clinical pneumonia, due both to fewer misclassified cases and to a reduction in cases that are caused by malaria. This prediction is supported by data from Kilifi, Kenya, where surveillance data on both malaria parasitaemia and pneumonia are available. Between 2002 and 2011, the prevalence of malaria parasitaemia in children in Kilifi dropped steeply from ∼35% to 2% whereas the incidence of severe or very severe WHO clinical pneumonia dropped by ∼50%, from ∼2 per 1000 population to 1 per 1000 population [29, 30]. Our model predicts a decline of ∼40% in clinical pneumonia cases (30% due to misclassified malaria and 10% directly attributable to malaria). Thus, the decline in clinical pneumonia in Kilifi is largely explained by the reduction in malaria.

The assumptions that underlie our estimation of PPV in PERCH merit evaluation. By using chest radiography as our reference standard, we assumed that CXR is 100% specific. Reasonable evidence to support this assumption exists at one of our sites [6]; the only convincing exception to this rule is pulmonary oedema, which may occur in late stages of severe malaria or malaria anaemia [31, 32]. The assumption that the CXR result in true pneumonia cases is independent of malaria infection is probably also reasonable because, as far as we are aware, there is no pathophysiological evidence to suggest that malaria coinfection influences the development of radiological features of pneumonia. However, our assumption of 100% specificity of the WHO pneumonia definition among admissions that are negative for malaria parasitaemia is potentially open to greater question. In particular, our model does not account for loss of specificity due to other pathologic processes that can lead to a pneumonia-like presentation, including sepsis, anaemia, and kerosene poisoning [33].

The most important limitation of the study was the relatively low prevalence of malaria parasitaemia in the healthy populations from which the data were drawn. In both the Kenyan and Gambian study sites (Kilifi and Basse), malaria has declined significantly since the early 2000s [29, 34] whereas the Mali site (Bamako) has less malaria compared with other areas of the country [35]. This not only reduced the precision of our estimates of PPV in the PERCH study, but also meant that it was necessary to extrapolate beyond the observed data to estimate PPV in settings with more typical malaria prevalence. In particular, we assumed a linear relationship between the prevalence of malaria parasitaemia in the community and the incidence of malaria disease. No consensus yet exists on the appropriate form for this relationship, though a recent analysis suggests that it is approximately linear in children aged <5 years [36]. Although we cannot be sure of the direction of bias, a saturating relationship between malaria prevalence and incidence, as opposed to the assumed linear relationship, would mean that our PPV estimates are likely too low.

Our analysis of clinical features of pneumonia revealed that nasal flaring, crackles on chest auscultation, and lower chest wall indrawing are predictive of CXR-positivity among patients with both a clinical diagnosis of pneumonia and malaria parasitaemia. Given that CXRs are not routinely available to support the diagnosis of pneumonia in many malaria-endemic countries, it may be useful to give these features added weight in clinical decision-making.

This study highlights and quantifies the extent to which the common condition of malaria compromises the specificity of the WHO clinical definition of pneumonia. Ultimately, it may be possible to solve this problem through the implementation of better pneumonia diagnostics. Ultrasound that is carried out at the point of care is particularly promising, as it is less expensive than CXR and free from ionizing radiation [37]. However, for the foreseeable future, management of patients with symptoms of pneumonia and studies of pneumonia epidemiology must allow for uncertainty in the aetiology of pneumonia. A child who meets the criteria for severe pneumonia under the WHO 2013 classification and also tests positive for malaria parasitaemia should be treated for both severe pneumonia and severe malaria. This means they should receive both parenteral broad-spectrum antibiotics and parenteral artesunate, and both treatments should be started without delay [38, 39]. Similarly, a child who is diagnosed with non-severe pneumonia under the 2013 WHO classification and tests positive for malaria parasitaemia should be managed at home with oral amoxicillin and an oral antimalarial medication [14].

## Ethics approval

The study protocol was approved by the institutional review board or ethical review committee that was overseeing each site and at the Johns Hopkins Bloomberg School of Public Health. Parents or guardians of participants provided written informed consent.

## Supplementary Material

dyaf040_Supplementary_Data

## Data Availability

It is anticipated that the PERCH data will be made available through the ClinEpi data portal at https://clinepidb.org/ce/app/workspace/analyses/DS_1595200bb8 in the upcoming months. Meanwhile, access can be requested from Christine Prosperi at Johns Hopkins University (e-mail: cprospe1@jhu.edu).
