# Supplementary material for: Misclassification of malaria as pneumonia in children in sub-Saharan Africa
Source: Int J Epidemiol. 2025 Apr 10;54(2):dyaf040. doi: 10.1093/ije/dyaf040 (PMC11984459; doi:10.1093/ije/dyaf040)
Supplement: dyaf040_Supplementary_Data [file dyaf040_supplementary_data.docx]

Supplementary Materials for

**Misclassification of malaria as pneumonia in children in sub-Saharan Africa.**

**Authors**

Christian Bottomley, Alice Kamau, Juliet O. Awori, Amanda J. Driscoll, Daniel E. Park, Samba O. Sow, Milagritos D. Tapia, Karen L. Kotloff, Bernard E. Ebruke, Martin Antonio, Stephen R. C. Howie, Richard J. Hayes, J. Anthony G. Scott

Correspondence to: [christian.bottomley@lshtm.ac.uk](mailto:christian.bottomley@lshtm.ac.uk)

Table of Contents

[Collection of clinical data 2](#_Toc176686851)

[Laboratory tests and procedures 2](#_Toc176686852)

[Logistic regression modelling 2](#_Toc176686853)

[Derivation of the formula used to estimate positive predictive value (PPV) in PERCH 3](#_Toc176686854)

[Figure S1: Sensitivity analysis 5](#_Toc176686855)

[Table S1: Factors associated with CXR positivity in malaria slide-positive WHO pneumonia cases 6](#_Toc176686856)

[Code for conducting logistic regression analyses in Stata 7](#_Toc176686857)

[References 8](#_Toc176686858)

# Collection of clinical data

Patients underwent a standardized clinical assessment that included medical history, respiratory findings, World Health Organisation (WHO) danger signs, comorbidities, and possible risk factors. Cases were identified as children aged 1-59 months hospitalized with severe or very severe pneumonia. Severe pneumonia was defined by cough or difficulty breathing with lower chest wall indrawing; very severe pneumonia was defined by cough or difficulty breathing and at least one danger sign (i.e. central cyanosis, difficulty breastfeeding/drinking, vomiting everything, convulsions, lethargy, reduced consciousness, or head nodding)^1^.

Cases were excluded if they had been discharged as a PERCH case within the past 30 days; or did not reside in the study catchment area. In addition, cases presenting at the enrolment site hospital within 14 days of hospitalisation were excluded. This was done to ensure the exclusion of hospital-acquired infections, as the focus of PERCH was on community-acquired pneumonia. Finally, children with pneumonia and wheeze were excluded if, following bronchodilator therapy, their lower chest wall indrawing resolved.

A chest x-ray (CXR) was performed on all cases. CXRs were read and adjudicated according to WHO-standardized interpretation procedures by trained readers who were blinded to the clinical or laboratory findings^2^. CXR positivity was defined by evidence of alveolar consolidation or any other infiltrate on a CXR performed up to 72 hours after presentation ^2,3^.

# Laboratory tests and procedures

Laboratory analysis of blood, lung aspirate, pleural aspirate, oro- and naso-pharyngeal samples, and urine is described elsewhere^4^. To ascertain malaria infection status, blood samples from cases and controls were analysed for the presence of malaria parasites using microscopy of Giemsa-stained thick and thin films. A blood sample was classified as positive if malaria parasites were detected at any density^4–6^. C-reactive protein (CRP) was measured in cases only, and only in instances where a serum specimen was collected.

# Logistic regression modelling

We used logistic regression to investigate the performance of various signs and symptoms as predictors of CXR positivity among children who tested slide-positive for malaria and met the WHO clinical case definition for pneumonia. The following variables were included in the model as potential confounders: sex, age at recruitment, month of recruitment, mother’s level of education and site. Analyses were conducted using Stata 13.1 (StataCorp, College Station, USA).

#

# Derivation of the formula used to estimate positive predictive value (PPV) in PERCH

The determination of the positive predictive value relies on three assumptions:

1. The WHO definition of pneumonia is 100% specific among malaria-slide negative hospital admissions.
2. CXR positivity is 100% specific for true pneumonia.
3. The sensitivity of CXR findings is independent of the presence of malaria parasitaemia.

Let $m$, $n$ and $x$ denote malaria parasitaemia, true pneumonia status, which is unobserved, and CXR result – for example, $m^{+}$ denotes positive for malaria parasitaemia.

The positive predictive (PPV) is the sum of the proportion of true pneumonia cases with malaria and the proportion without malaria:

$PPV= p(n^{+}, m^{-})+ p(n^{+}, m^{+})$.

We want to show that given Assumptions 1-3, PPV is equivalent to

$PPV = p\left( m^{-} \right)+ p\left( x^{+}, m^{+} \right) p\left( m^{-} \right)/p\left( x^{+}, m^{-} \right)$.

From Assumption 1, $p\left( n^{-}, m^{-} \right)=0$ and therefore $p\left( n^{+}, m^{-} \right)= p\left( m^{-} \right).$

From the definition of conditional probability:

$p\left( n^{+}, m^{+} \right)=$ $p\left( x^{+},n^{+}, m^{+} \right)$/$p\left( x^{+} \right|{n^{+}, m}^{+}).$

Furthermore, from Assumption 2

$p\left( x^{+},n^{-}, m^{+} \right)=0$and therefore $p\left( x^{+},n^{+}, m^{+} \right)=p\left( x^{+}, m^{+} \right)$.

Finally,

$p\left( x^{+} \right|{n^{+}, m}^{+})= p\left( x^{+} \right|{n^{+}, m}^{-})$ [Assumption 3]

$= p\left( x^{+},n^{+}, m^{-} \right)/p\left( n^{+}, m^{-} \right)$

$= p\left( x^{+},n^{+}, m^{-} \right)/p\left( m^{-} \right)$ [Assumption 1]

$= p\left( x^{+}, m^{-} \right)/p\left( m^{-} \right)$. [Assumption 2]

# Figure S1: Sensitivity analysis showing the impact of halving and doubling the incidence of true pneumonia relative to the incidence in the Pneumonia Etiology Research for Child Health (PERCH) study.

# Table S1: Factors associated with chest X-ray (CXR) positivity in malaria slide-positive clinical pneumonia cases

| **Clinical presentation** | **CXR +ve**  **(n/N)** | **CXR -ve**  **(n/N)** | **Crude OR**  **(95% CI)** | **Adjusted OR***  **(95% CI)** | ***P* value** |
| --- | --- | --- | --- | --- | --- |
| Head nodding | 0/18 | 2/59 | NA | NA | NA |
| Central cyanosis | 0/18 | 1/59 | NA | NA | NA |
| Pedal edema | 1/18 | 0/59 | NA | NA | NA |
| Lethargic | 6/18 | 28/59 | 0.55 (0.18, 1.67) | 0.16 (0.02, 1.06) | 0.058 |
| Temperature >38°C or history of fever | 9/18 | 38/59 | 0.55 (0.19, 1.61) | 0.22 (0.04, 1.10) | 0.065 |
| CRP cutoff 40 mg/L^a^ | 5/12 | 30/46 | 0.38 (0.10, 1.40) | 0.24 (0.03, 2.33) | 0.221 |
| Unable to feed | 2/18 | 11/59 | 0.55 (0.11, 2.73) | 0.26 (0.01, 4.89) | 0.366 |
| Grunting | 3/18 | 11/59 | 0.87 (0.21, 3.55) | 0.97 (0.09, 10.75) | 0.983 |
| Tachypnea | 15/18 | 43/59 | 1.86 (0.47, 7.29) | 1.23 (0.16, 9.37) | 0.841 |
| Vomiting everything | 2/18 | 5/57 | 1.06 (0.19, 5.79) | 1.70 (0.12, 24.09) | 0.695 |
| Hypoxia^b^ | 9/18 | 11/59 | 4.36 (1.41, 13.54) | 3.47 (0.62, 19.30) | 0.156 |
| **Nasal flaring** | **12/18** | **18/59** | **4.56 (1.48, 14.04)** | **5.93 (1.07, 32.83)** | **0.041** |
| **Crackles on chest auscultation** | **7/18** | **9/59** | **3.54 (1.08, 11.55)** | **13.10 (1.35, 127.37)** | **0.027** |
| **Lower chest wall indrawing** | **14/18** | **21/59** | **6.33 (1.85, 21.72)** | **18.14 (1.87, 175.84)** | **0.012** |

*Odds ratios adjusted for sex, age, month of recruitment, mother’s education and site. Bold indicates p-value <0.05. Abbreviations: CXR, chest x-ray; CI, confidence interval; OR, odds ratios; NA, omitted because adjusted odds ratios cannot be calculated when there are no cases in one of the groups; ^a^C-reactive protein (CRP) level; ^b^Hypoxia was defined as oxygen saturation <92%, or oxygen requirement (if on oxygen and room air saturation not available).

# Code for conducting logistic regression analyses in Stata

/*=====================================================================

Program Name: Misclassification of malaria as pneumonia

Author......: Alice Kamau

Date........: 13 May 2016

Purpose.....; To perform analyses for etiology by malaria

=====================================================================*/

capture log close

clear all

set more off

set memory 4g

********************************************

cd "~"

log using "logs/misclassification_analysis.txt", replace

* read the data

import excel "data/PERCH site data (280623).xlsx", clear firstrow

/******************************

Manuscript analysis

******************************/

egen _sex = group(sex),label

egen site = group(_site), label

egen agecat = group(_agecat), label

* descriptives (Table 1)

tab group _sex, row chi //sex

tabstat _agem, s(mean sd p50 p25 p75 N) by(group) //age

tabstat _zwei, s(mean sd p50 p25 p75 N) by(group) //Weight for age z score

tabstat lrccbca, s(mean sd p50 p25 p75 N) by(group) //Hemoglobin

tabstat lrccbcg , s(mean sd p50 p25 p75 N) by(group) //white blood count

tabstat lrccbch, s(mean sd p50 p25 p75 N) by(group) //Neutrophils

* recode age in months

gen age_cat = cond(age_m >12, 1, 0)

label define age_cat 0 "< 1 year" 1 ">=1 year"

label value age_cat age_cat

* CXR+ PPV (Table 2)

cs cxrpos lrcmalrs

cs cxrpos lrcmalrs if elcvspn == 0

cs cxrpos lrcmalrs if elcvspn == 1

cs cxrpos lrcmalrs if age_cat == 0

cs cxrpos lrcmalrs if age_cat == 1

* SI Table

keep if _casecont == 1 & lrcmalrs == 1

tab motheduc,m

tab motheduc _site,m

* univariable regression

foreach var of varlist elchead elccentr csapeded _mpconvul _lethargic elevtemp _crp elcfeed csagrunt _tachypnea elcvom _hypox csanflar _crack elclcwi {

logistic cxrpos i.`var'

}

* multivariable regression

foreach var of varlist elchead elccentr csapeded _mpconvul _lethargic elevtemp _crp elcfeed csagrunt _tachypnea elcvom _hypox csanflar _crack elclcwi {

tab cxrpos `var', matcell(temp)

di temp[2,2]

di temp[2,1]

di temp[1,2]

di temp[1,1]

if (temp[2,1]!=0 & temp[2,2]!=0 & temp[1,1]!=0 & temp[1,2]!=0) {

logistic cxrpos i.`var' i.site i.agecat i.enrmonth i._sex i.motheduc

lsens, gensens(sensitivity) genspec(specificity) all replace saving(lsens_`var', replace)

estat classification, cutoff(0.25)

}

}

# References

1. Scott, J. A. G. *et al.* The definition of pneumonia, the assessment of severity, and clinical standardization in the Pneumonia Etiology Research for Child Health study. *Clin Infect Dis* **54 Suppl 2**, S109-16 (2012).

2. Cherian, T. *et al.* Standardized interpretation of paediatric chest radiographs for the diagnosis of pneumonia in epidemiological studies. *Bull World Health Organ* **83**, 353–359 (2005).

3. Fancourt, N. *et al.* Standardized Interpretation of Chest Radiographs in Cases of Pediatric Pneumonia From the PERCH Study. *Clin Infect Dis* **64**, S253–S261 (2017).

4. Driscoll, A. J. *et al.* Standardization of Laboratory Methods for the PERCH Study. *Clin Infect Dis* **64**, S245–S252 (2017).

5. Hammitt, L. L. *et al.* Specimen collection for the diagnosis of pediatric pneumonia. *Clin Infect Dis* **54 Suppl 2**, S132-9 (2012).

6. Murdoch, D. R. *et al.* Laboratory methods for determining pneumonia etiology in children. *Clin Infect Dis* **54 Suppl 2**, S146-52 (2012).
